# Inertial Measurement Units for Clinical Movement Analysis: Reliability and Concurrent Validity

**DOI:** 10.3390/s18030719

**Published:** 2018-02-28

**Authors:** Mohammad Al-Amri, Kevin Nicholas, Kate Button, Valerie Sparkes, Liba Sheeran, Jennifer L Davies

**Affiliations:** 1 School of Healthcare Sciences, College of Biomedical and Life Sciences, Cardiff University, Cardiff CF24 0AB, Wales, UK; NicholasKA@cardiff.ac.uk (K.N.); ButtonK@cardiff.ac.uk (K.B.); SparkesV@cardiff.ac.uk (V.S.); SheeranL@cardiff.ac.uk (L.S.); DaviesJ@cardiff.ac.uk (J.L.D.); 2 Arthritis Research UK Biomechanics and Bioengineering Centre, Cardiff University, Cardiff CF10 3AXB, Wales, UK; 3 Cardiff and Vale University Health Board, Heath Park, Cardiff CF14 4XW, Wales, UK

**Keywords:** inertial measurement units, motion analysis, kinematics, gait, functional activity, repeatability, reliability, biomechanics

## Abstract

The aim of this study was to investigate the reliability and concurrent validity of a commercially available Xsens MVN BIOMECH inertial-sensor-based motion capture system during clinically relevant functional activities. A clinician with no prior experience of motion capture technologies and an experienced clinical movement scientist each assessed 26 healthy participants within each of two sessions using a camera-based motion capture system and the MVN BIOMECH system. Participants performed overground walking, squatting, and jumping. Sessions were separated by 4 ± 3 days. Reliability was evaluated using intraclass correlation coefficient and standard error of measurement, and validity was evaluated using the coefficient of multiple correlation and the linear fit method. Day-to-day reliability was generally fair-to-excellent in all three planes for hip, knee, and ankle joint angles in all three tasks. Within-day (between-rater) reliability was fair-to-excellent in all three planes during walking and squatting, and poor-to-high during jumping. Validity was excellent in the sagittal plane for hip, knee, and ankle joint angles in all three tasks and acceptable in frontal and transverse planes in squat and jump activity across joints. Our results suggest that the MVN BIOMECH system can be used by a clinician to quantify lower-limb joint angles in clinically relevant movements.

## 1. Introduction

Assessment of movement patterns during functional activities such as walking and squatting, and during sporting manoeuvres such as jumping, is a cornerstone of musculoskeletal physiotherapy. Commonly, the physical examination involves observation by the clinician and completion of clinician- or patient-rated scales [1]. However, it is challenging to accurately evaluate multiple joints of both legs in multiple planes of movement when an individual is performing a dynamic functional activity, which often occurs at speed. Three-dimensional optoelectronic (camera-based) motion systems can be used to provide comprehensive, objective measurements [2], but this typically requires the patient to attend a specialised movement analysis laboratory. The equipment within these laboratories is expensive, non-portable, and requires a high level of technical expertise and a lengthy calibration process. The use of these systems is therefore not widespread in clinical practice, and clinicians typically do not have access to objective biomechanical information for assessing patient performance. A more rigorous approach to quantifying joint movement in the clinic is required.

A potential solution to this problem is inertial measurement units (IMUs), which could be used in clinical settings to objectively measure movement patterns during functional activities. An IMU is comprised of accelerometers, gyroscopes, and magnetic sensors combined with a fusion algorithm, for example a Kalman filter [3,4,5]. It can be attached to a body segment to estimate the movement of that segment in space. When combined with other IMUs on adjacent body segments, the kinematics of movements can be calculated [6,7,8,9,10,11,12]. IMU motion-capture systems are portable and less expensive than traditional camera-based motion-capture systems. The validity of joint kinematics calculated with IMU systems has been confirmed with respect to optoelectronic systems [10,11,13].

Commercial IMU systems are increasingly available, from companies such as Xsens Technologies B.V., (Enschede, The Netherlands), Shimmer Sensing (Dublin, Ireland), BioSyn Systems (Surrey, BC, Canada), I Measure U (Auckland, New Zealand), and APDM Wearable Technologies (Portland, OR, USA). The Xsens MVN BIOMECH system is composed of wireless motion IMUs (called MTw2 sensors) and native biomechanical protocols, and estimates three-dimensional joint kinematics [14]. The accuracy of the orientation information provided by an individual IMU [15,16,17] and the joint angles provided by MVN BIOMECH software [18,19] have been reported. Robert-Lachaine et al. [18] and Zhang et al. [19] compared joint angles obtained from MVN BIOMECH software against those obtained from a biomechanical model associated with the Optotrak camera-based motion analysis system. During walking and stair climbing, the mean differences in lower-limb joint angles between the two systems were between 1.4° and 6.7°, and the coefficient of multiple correlation (CMC) was between 0.39 and 0.99 [19]. In more complex manual handling tasks, errors due to technology were < 5° and CMC was 0.79 to 0.97 [18]. This suggests that the MVN BIOMECH system is able to quantify joint kinematics during complex functional movements. In these studies, the motion analysis systems were operated by individuals who were experienced in performing movement analysis experiments, and not by clinicians. However, if an IMU system is to be used in a clinical setting, it is most likely to be used by a clinician with little or no experience of collecting biomechanical data. At present it is not clear if undergoing brief training using only the guides provided by the manufacturer is sufficient to enable a clinician to collect accurate and reliable biomechanical information using a commercially available IMU system. Additionally, the tasks evaluated in previous studies were limited to walking and stair climbing [19], and manual handling tasks [18]. To facilitate transfer of this technology to the clinic it is necessary to quantify the validity and reliability of the Xsens MVN BIOMECH system when used by a clinician, and for clinically relevant functional movements that may involve a greater range of motion or dynamic movements, such as squat and jump.

The primary aim of this study was to determine the reliability of joint angular kinematics provided by the Xsens MVN BIOMECH system when used by a clinically experienced musculoskeletal physiotherapist (MSKP) with no experience of using IMUs across different sessions (i.e., within-rater, between-session repeatability). Secondary aims were (1) to determine the reliability of joint angular kinematics provided the Xsens MVN BIOMECH system when used by an experienced MSKP and an experienced clinical movement scientist (i.e., between-rater, within-session repeatability); and (2) to determine the concurrent validity (agreement) of joint angular kinematics provided the Xsens MVN BIOMECH system when used by an experienced MSKP against data collected using a gold-standard camera-based motion capture system (VICON motion analysis system, Oxford Metrics Group Ltd., Oxford, UK). Validity and reliability were evaluated during walking, squatting and jumping to cover a larger range of motion (squat, jump) and a more dynamic movement (jump) than previously investigated.

## 2. Materials and Methods

### 2.1. Research Participants and Setting

The study was carried out in the Research Centre for Clinical Kinaesiology at Cardiff University. The study was conducted as part of the Arthritis Research UK Biomechanics and Bioengineering Centre at Cardiff University, and was approved by the Wales Research Ethics Committee 3 (10/MRE09/28). The required sample size (*n* = 16) was determined according to the recommendation of Walter et al. (Table II, [20]), using *α* = 0.05, *β* = 0.2, *ρ*_0_ =0.2 and *ρ*_1_ = 0.7. To account for an anticipated attrition of 40% over the repeated sessions, the target sample size was increased to 27. A convenience sample of healthy participants was recruited using the following criteria: age between 18 and 60 years; healthy with no known neurological, cardiovascular, or musculoskeletal condition. Written informed consent was obtained prior to participation.

### 2.2. Raters

The Xsens MVN BIOMECH system was used by two raters. Rater 1 (MSKP) had 13 years experience as a qualified physiotherapist and 5 years experience as a specialist knee physiotherapist, but no prior experience of biomechanical motion capture. Rater 2 (clinical movement scientist) had 10 years experience performing biomechanical motion capture studies in laboratory settings. The two raters received the same information and instructions for use of the Xsens MVN BIOMECH system. This consisted of self-training using the MVN user manual [21] and online tutorials (https://tutorial.xsens.com/) and a half-day training session provided by Xsens Technologies at Cardiff University.

### 2.3. Experimental Protocol

Each participant underwent two movement analysis sessions, approximately 1 week apart (mean ± standard deviation, 4 ± 3 days). In the first session, anthropometric measurements were taken by the MSKP from the right lower limb with the participant in a standing posture. Body mass was recorded using SECA scales (Birmingham, UK). These measurements were only taken in the first session. Apart from this, the two sessions were identical and proceeded as follows:

At the start of each session, 16 retroreflective markers (14-mm diameter) were placed on the participant according to the Plug-in-Gait lower-body model (Vicon Motion Systems, Oxford Metrics Group Ltd.). In every session, retroreflective markers were placed by the MSKP using standardised palpation methods on anatomical landmarks [22]. Retroreflective markers were always attached prior to placement of any IMU, and were held in position with medical grade double-sided adhesive tape.

Seven MTw2 trackers (Xsens Technologies) were then placed in accordance with Xsens instructions [21]. MTw2 trackers were placed by either rater 1 (MSKP) or rater 2 (clinical movement scientist). The order of the raters was randomised across participants, but consistent within participants across sessions. MTw2 trackers were secured using elasticated Velcro straps on each upper thigh (centrally and halfway between the greater trochanter and lateral epicondyle of the knee), each lower leg (proximal medial surface of the tibia), the dorsum of each foot and one centrally over the sacrum. Each lower-limb tracker was placed between the two outermost layers of the strap and attached to the Velcro of the inner layer to secure its position and minimise any movement. The sacral tracker was placed directly over the sacrum with the upper border of the sensor aligned centrally between the two posterior superior iliac spines. The sacral sensor was held in position with medical grade double-sided adhesive tape. Care was taken not to interfere with the reflective markers.

Each participant performed eight repetitions of each of the following three activities: over-ground walking, squatting, and vertical jumping. Prior to performing each activity the participant was provided a demonstration by the MSKP, and was allowed to ask any questions. All activities were performed at a self-selected speed to best mimic performance of these activities in a clinical setting. The order of the activities was randomised across participants, but consistent within participants and across sessions. Each walking trial consisted of a walk in a straight line across the laboratory (approximately 8 m). Squat depth was standardised to prevent occlusion of the reflective markers on the anterior superior iliac spines at low squat depths. This was done using a plinth placed behind the participant at the height of the knee joint line plus 5 cm, which prevented the participant from squatting past this depth. Once all activities were completed, the MTw2 trackers were removed by the same rater who had placed them. The participant rested for at least 10 min to allow any strap marks on the skin to disappear, and then the MTw2 trackers were placed by the other rater. The retroreflective markers were not moved during this rest period. The participant then repeated the activities in the same order as in the first half of the session.

### 2.4. Data Collection

Kinematic data were collected at 120 Hz using a ten-camera VICON MX3+ motion analysis system (Oxford Metrics Group Ltd.) and 60 Hz using the Xsens MVN BIOMECH system (Xsens Technologies). The systems were synchronised using a trigger sent from the MVN BIOMECH studio software (Version 4.4) to VICON Nexus software (Version 1.8). Prior to beginning the tasks, the participant was asked to stand in a static N-pose, as per the instructions in the MVN BIOMECH user manual [21]. This was maintained for ~ 30 s. At the start of this period of quiet stance, the MTw2 trackers were calibrated within the MVN BIOMECH software. During this process the software establishes the relation between segment and tracker orientations [23,24]. Once this was complete (~10 s), the remaining duration of the quiet stance was synchronously recorded by the two systems. These data were used offline in VICON Nexus software as the static anatomical calibration trial for the Plug-In-Gait model.

### 2.5. Data Processing

Data collected using the MVN BIOMECH system were exported as an mvnx file. For data collected using the VICON system, reconstruction and auto-labelling of marker trajectories was first performed in VICON Nexus software. Each trial was then visually inspected and unmarked trajectories were manually labelled. Gaps in trajectories of up to 10 samples were joined with linear interpolation filtered with a quintic spline filter (Woltring; mean square error of 15). The Plug-in Gait pipeline was then implemented in VICON Nexus software, and resulting kinematic data were exported as a c3d file. Plug-in Gait is the commercial name for the implementation of what is commonly called the conventional gait model [22,25,26,27].

Custom analysis scripts were created in Matlab software (version 2015a; The MathWorks Inc., Natick, MA, USA) and used to perform subsequent analyses. Hip, knee and ankle joint angles calculated by MVN BIOMECH software and VICON Nexus software were extracted from the mvnx and c3d files, respectively. In VICON, positive joint values indicate flexion, adduction and internal rotation in the sagittal, frontal and transverse planes, respectively. In MVN BIOMECH, positive joint angles indicate flexion, abduction and internal rotation, respectively. Frontal plane joint angles from MVN BIOMECH were therefore inverted before any further analysis. Data were filtered with a 6-Hz low-pass fourth-order Butterworth filter. Vicon data were resampled to 60 Hz using the resample function in Matlab. No other post-processing was performed on the joint angles provided by the two systems. Although the systems were synchronised, we observed discrepancies in the duration of the files recorded by the two systems. Due to the uncertainty as to whether this failure of synchronisation occurred at the start or the end of the recording, the mvnx and c3d files were aligned using the ‘aligndata’ function within Matlab performed on the sagittal plane knee angle.

Movement cycles were defined using data from VICON, as described in Table 1. For each movement cycle, the following variables were quantified for hip, knee and ankle joints on both sides of the body in the sagittal, frontal and transverse planes: minimum angle, maximum angle, range of motion. For walking trials, the joint angle at heel strike was also identified. The minimum and maximum angle, the range of motion and the angle at heel strike represent discrete, clinically relevant events in the movement cycles [28,29,30,31]. If the MVN BIOMECH system is to be transferred into the clinic it is important that it can accurately and reliably measure these angles at these time points.

### 2.6. Data Analysis

Data analysis was carried out in Matlab software for each activity (walk, jump, squat), each joint (hip, knee, ankle), each plane of movement (sagittal, frontal, transverse) and each side of the body (left, right). For each walk trial, a single stride from the middle of the trial was used for analysis. This gave eight strides for analysis, alongside eight repetitions of squat and jump. The descriptive analysis included mean, standard deviation, difference, and 95% confidence interval of difference of each outcome measure. These were calculated for each participant as an average of all repetitions, before being averaged across all participants. Reliability and validity were quantified for each activity, each joint, and each plane according to the COnsensus-based Standards for the selection of health Measurement INstrument (COSMIN) standards [33,34] as detailed below.

#### 2.6.1. Reliability

Within-rater, between-session reliability was evaluated for rater 1 (MSKP) by comparing data from session 1 and session 2. Between-rater (within-session) reliability was evaluated for session 1 by comparing data from rater 1 (MSKP) and rater 2 (clinical movement scientist). Reliability was quantified using single ICC. For this calculation, the two-way random effects model was used, with a confidence of 95%. ICC was interpreted according to Shrout and Fleiss [35] where ICC ≥ 0.75 indicates excellent repeatability, ICC 0.4–0.74 indicates fair-to-high repeatability, and ICC ≤ 0.39 indicates poor repeatability. Standard error of measurement (SEM) was also calculated using Equation (1) [36]:

(1) $$\mathrm{SEM}=\mathrm{SD}\times \sqrt{1-\mathrm{ICC}}$$

where SD refers to the standard deviation. SEM was used to evaluate absolute reliability and provide information on variability over repeated measurements [37]. Absolute reliability indicates the reliability of scores within individual participants on different occasions, and was considered excellent if SEM < 3.0° and acceptable if SEM < 5° [38].

#### 2.6.2. Concurrent Validity

Concurrent validity was evaluated for rater 1 (MSKP) using data from session 1. For each activity (walk, jump, squat), each joint (hip, knee, ankle), each plane of movement (sagittal, frontal, transverse) and each side of the body (left, right), MVN BIOMECH and VICON data were compared using the difference in minimum angle, maximum angle, and range of motion (Δmin *θ*, Δmax *θ*, and ΔROM, respectively), the coefficient of multiple correlation (CMC [10,25,39,40]), and the linear fit method (LFM [41]). In addition, for walking trials, MVN BIOMECH and VICON data were compared using the difference in the angle at heel strike (Δ@HS).

Delta values indicate the similarity of the two signals at clinically relevant points in the movement cycle. The CMC and LFM indicate the similarity of the two signals across the full movement cycle. The CMC was calculated using the formula provided in Ferarri et al. [10]. When the range of motion of the two waveforms is comparable to the offset between them, the CMC is not a real number. Following Ferarri et al. [10,39], CMC was calculated before and after the offset was removed from the two signals. For each signal (MVN BIOMECH and VICON), offset was calculated as the mean of the signal over the entire movement cycle. This value was then subtracted from the signal, giving a signal with zero offset. The CMC before and after offset removal is referred to as CMC_1_ and CMC_2_ respectively. CMC is reported only for joints and planes where all values are real numbers [10]. The LFM gives three parameters: α1 indicates the scaling factor, α indicates the scalar addition (i.e., shift or offset) and *R*^2^ indicates the strength of the linear relation between the two signals [41]. Concurrent validity was considered excellent if CMC and *R*^2^ > 0.75, fair-to-high if CMC and *R*^2^ 0.4–0.74, and poor if CMC and *R*^2^ < 0.39.

## 3. Results

### 3.1. Demographics and Descriptive

Twenty-six participants (mean ± standard deviation age: 35.2 ± 8.4 years; height: 162.0 ± 32.9 cm; body mass: 71.6 ± 12.8 kg; body mass index: 25.3 ± 3.8 kg/m^2^) were enrolled in the study and completed the first session. These individuals were all recruited in the first wave of recruitment. Although this was one below our target recruitment of 27, at the end of the first wave of our recruitment it was clear that our attrition of 4% was well below the allowed 40%, and so a 27th participant was not sought. One participant (4%) did not return for the second session. For the remaining 25 participants, the duration between sessions was 4 ± 3 days. For a second participant, data were not processed for session 1 and for a third participant, data were not processed for the jump activity for session 1, rater 1. In both cases this was because clothing obscured the right thigh marker. Between-session reliability of MVN BIOMECH data was therefore evaluated for 24 participants, apart from the jump where it was evaluated for 23 participants. Similarity of MVN BIOMECH and VICON data was evaluated using data from session 1, and was therefore evaluated for 25 participants. Results were similar for left and right sides of the body, and only results for the left side of the body are presented. The mean ± SD speed of walking was 1.40 ± 0.14 m/s in session 1 and 1.39 ± 0.13 m/s in session 2. The mean ± SD duration of squat was 2.25 ± 0.60 s in session 1 and 2.18 ± 0.62 s in session 2. The mean ± SD duration of jump was 1.21 ± 0.55 s in session 1 and 1.13 ± 0.38 s in session 2.

### 3.2. Reliability

**Figure 1:**
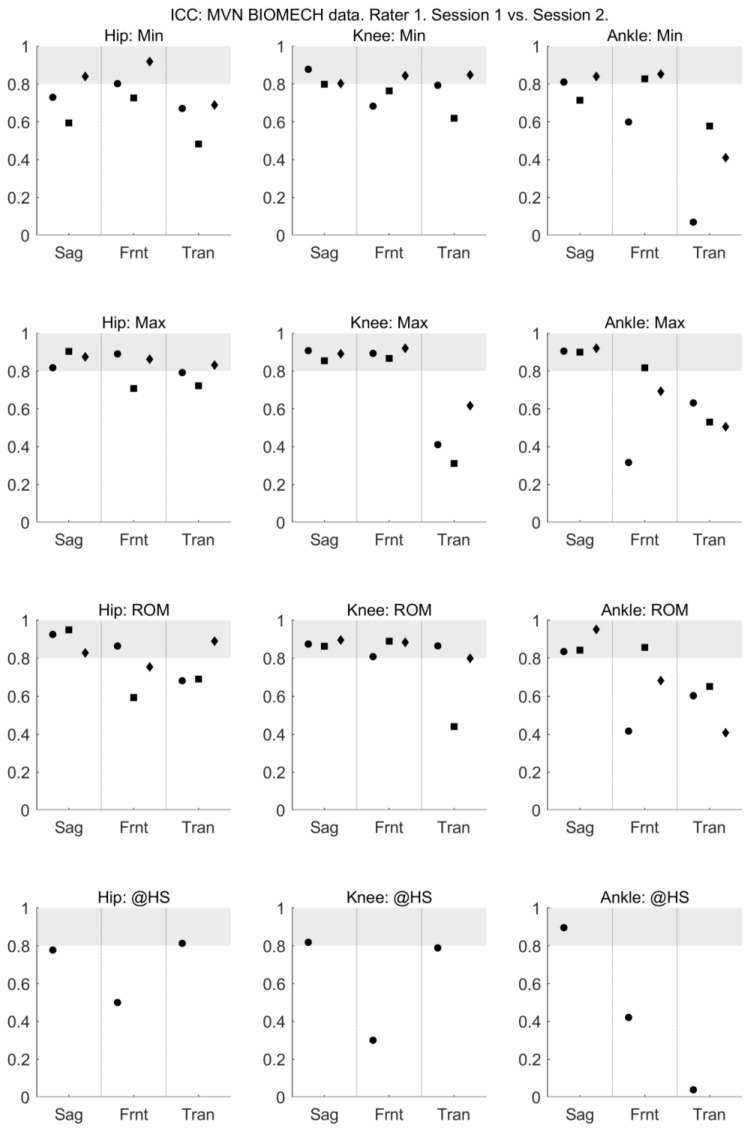
Between-session (within-rater) intraclass correlation coefficient of MVN data collected by rater 1 (musculoskeletal physiotherapist) in sessions 1 and 2. Intraclass correlation coefficient is shown for the minimum angle (min; **top row**), maximum angle (max; **second row**), range of motion (ROM; **third row**) and angle at heel strike (@HS; **bottom row**) for the hip (left-most column), knee (centre column) and ankle (right-most column) joints in the sagittal (Sag), frontal (Frnt) and transverse (Tran) planes of movement. The three data points in each plane correspond to the walk (left-most point; circle), squat (centre point; square) and jump (right-most point; diamond) tasks. Grey shading indicates values considered to indicate excellent reliability.

**Figure 2:**
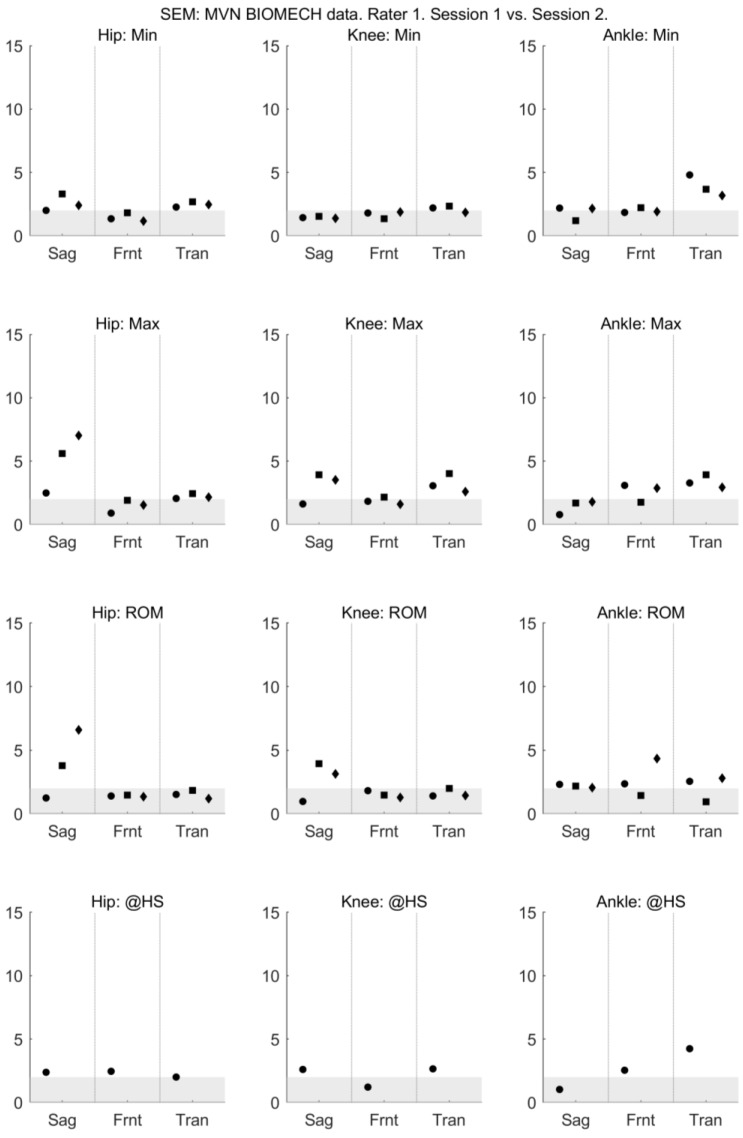
Between-session (within-rater) standard error of measurement of MVN data collected by rater 1 (musculoskeletal physiotherapist) in sessions 1 and 2. Standard error of measurement is shown for the minimum angle (min; **top row**), maximum angle (max; **second row**), range of motion (ROM; **third row**) and angle at heel strike (@HS; bottom row) for the hip (left-most column), knee (centre column) and ankle (right-most column) joints in the sagittal (Sag), frontal (Frnt) and transverse (Tran) planes of movement. The three data points in each plane correspond to the walk (left-most point; circle), squat (centre point; square) and jump (right-most point; diamond) tasks. Grey shading indicates values considered to indicate excellent reliability.

The mean and standard deviation of each measure, and the mean difference of each measure between days and raters are presented in Appendix A. For all activities and all joints, the between-session (within-rater) reliability of sagittal plane joint angles provided by MVN BIOMECH was high, with ICC between 0.6 and 0.95 (Figure 1). The between-session (within-rater) reliability of the frontal plane knee angle and transverse plane ankle angle at the heel strike was poor (Figure 1). The absolute between-session (within-rater) reliability of the MVN BIOMECH data was generally acceptable (SEM < 5°; Figure 2).

**Figure 3:**
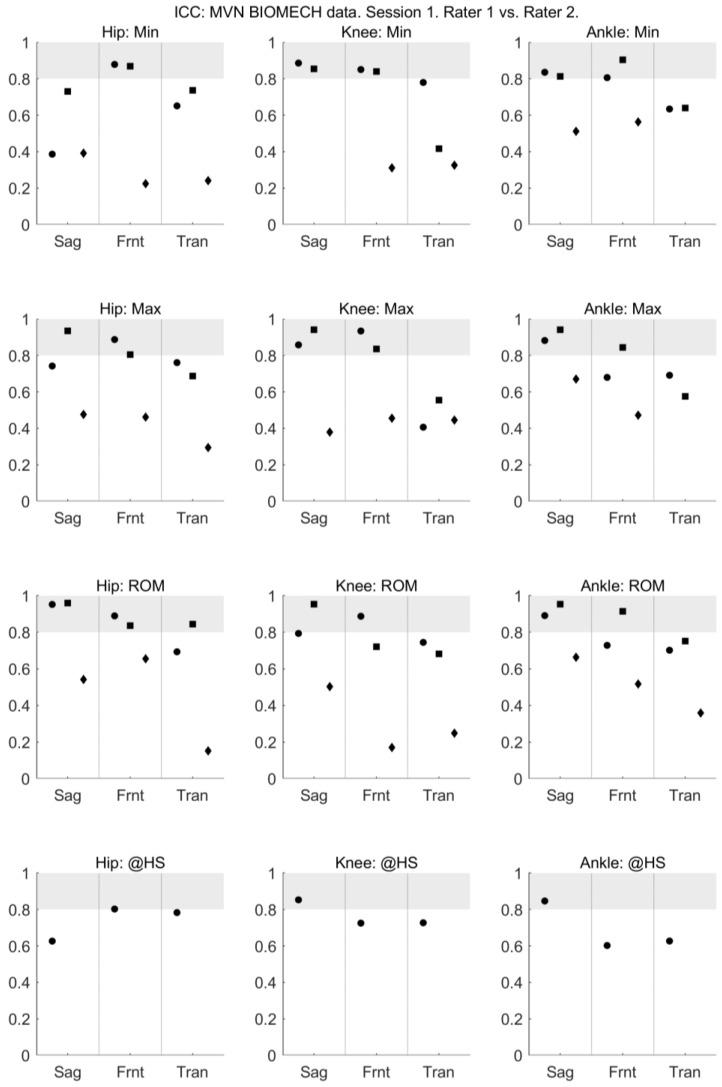
Between-rater intraclass correlation coefficient of MVN data collected by rater 1 (musculoskeletal physiotherapist) and rater 2 (clinical movement scientist) in session 1. Intraclass correlation coefficient is shown for the minimum angle (min; **top row**), maximum angle (max; **second row**), range of motion (ROM; **third row**) and angle at heel strike (@HS; **bottom row**) for the hip (left-most column), knee (centre column) and ankle (right-most column) joints in the sagittal (Sag), frontal (Frnt) and transverse (Tran) planes of movement. The three data points in each plane correspond to the walk (left-most point; circle), squat (centre point; square) and jump (right-most point; diamond) tasks. Grey shading indicates values considered to indicate excellent reliability.

**Figure 4:**
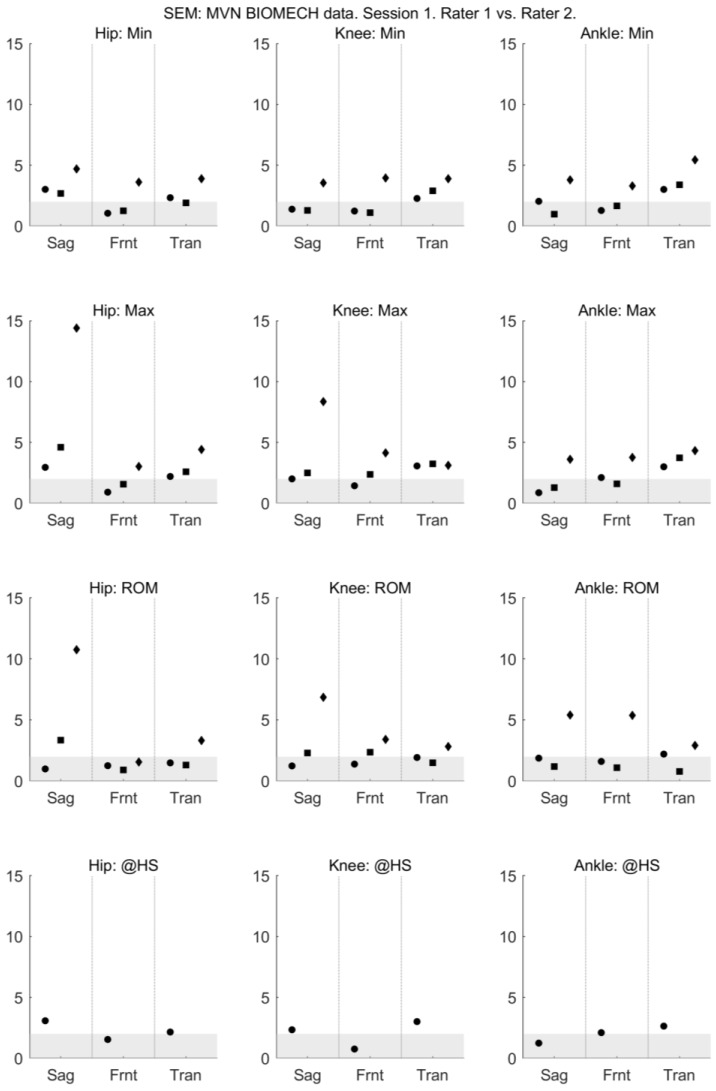
Between-rater standard error of measurement of MVN data collected by rater 1 (musculoskeletal physiotherapist) and rater 2 (clinical movement scientist) in session 1. Standard error of measurement is shown for the minimum angle (min; **top row**), maximum angle (max; **second row**), range of motion (ROM; **third row**) and angle at heel strike (@HS; **bottom row**) for the hip (left-most column), knee (centre column) and ankle (right-most column) joints in the sagittal (Sag), frontal (Frnt) and transverse (Tran) planes of movement. The three data points in each plane correspond to the walk (left-most point; circle), squat (centre point; square) and jump (right-most point; diamond) tasks. Grey shading indicates values considered to indicate excellent reliability.

For walking and squatting, the between-rater (within-session) reliability of all joint angles provided by MVN BIOMECH was acceptable, with ICC > 0.6 (Figure 3) and SEM < 5° (Figure 4) across all planes. For jumping, the between-rater (within-session) reliability of joint angles ranged from poor to excellent (Figure 3 and Figure 4).

### 3.3. Concurrent Validity

**Figure 5:**
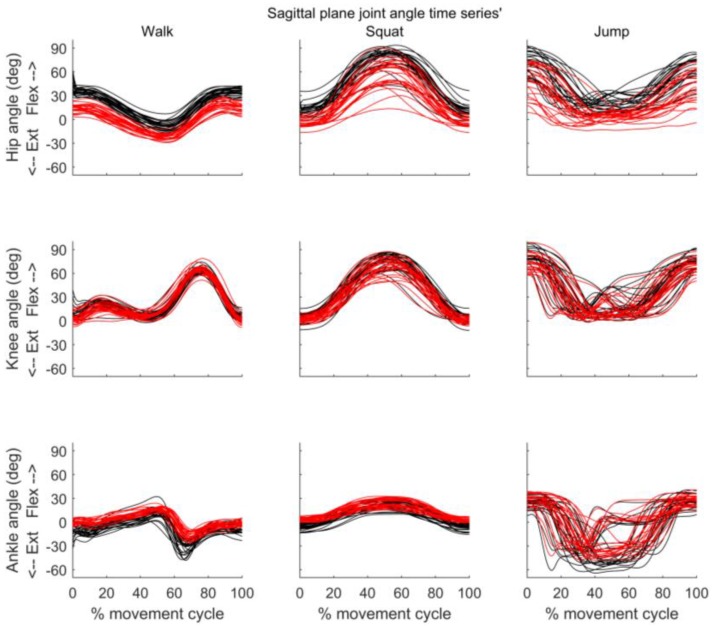
Sagittal plane joint angles throughout the movement cycle for each participant. Time series of hip (**top row**), knee (**middle row**) and ankle (**bottom row**) joint angles obtained from VICON (black) and MVN BIOMECH (red) systems for each participant during walk (**left**), squat (**centre**), and jump (**right**). Y-axis represents joint angles in degrees and X-axis represent the movement cycle in percentage.

**Figure 6:**
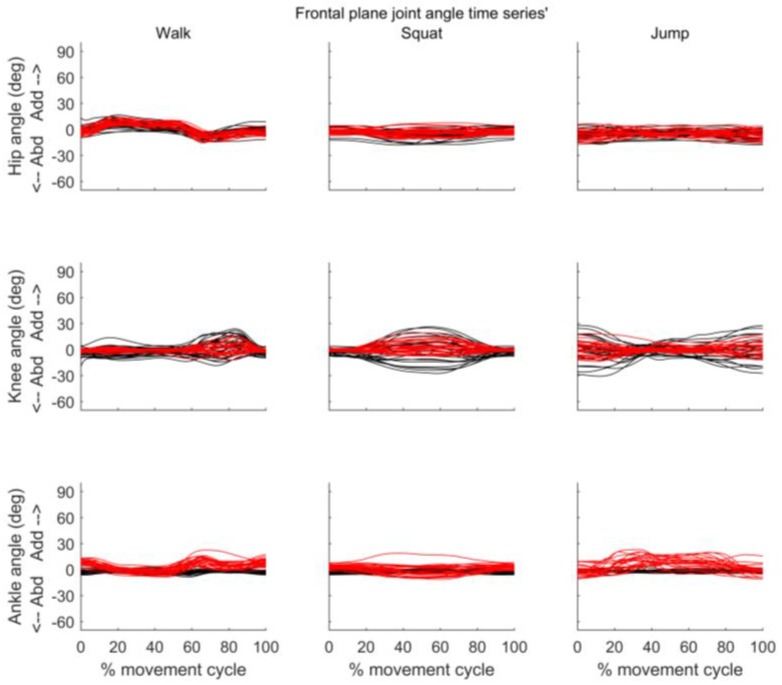
Frontal plane joint angles throughout the movement cycle for each participant. Time series of hip (**top row**), knee (**middle row**) and ankle (**bottom row**) joint angles obtained from VICON (black) and MVN BIOMECH (red) systems for each participant during walk (**left**), squat (**centre**), and jump (**right**). Y-axis represents joint angles in degrees and X-axis represent the movement cycle in percentage. Y-axis scale is the same as in Figure 5 to allow comparison.

**Figure 7:**
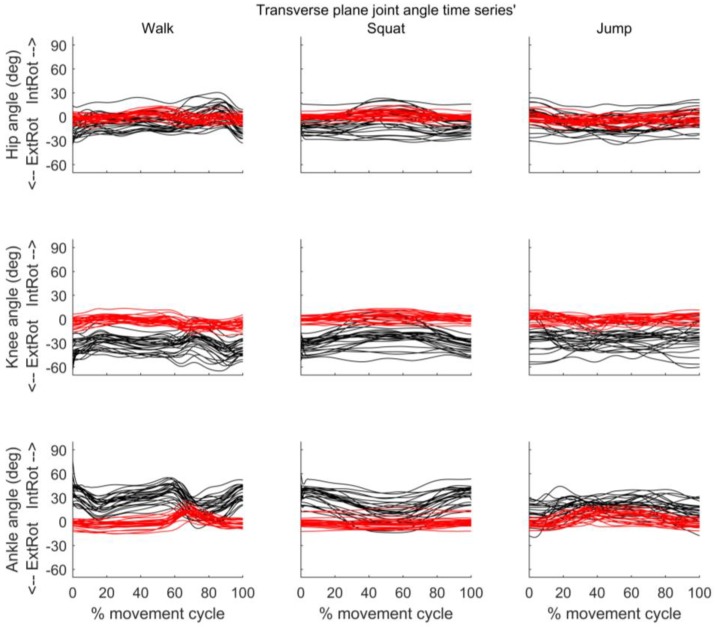
Transverse plane joint angles throughout the movement cycle for each participant. Time series of hip (**top row**), knee (**middle row**) and ankle (**bottom row**) joint angles obtained from VICON (black) and MVN BIOMECH (red) systems for each participant during walk (**left**), squat (**centre**), and jump (**right**). *Y*-axis represents joint angles in degrees and *X*-axis represent the movement cycle in percentage. *Y*-axis scale is the same as in Figure 5 and Figure 6 to allow comparison.

Figure 5, Figure 6 and Figure 7 present the joint angle time series’ obtained from MVN BIOMECH and VICON systems for each participant during walk, squat, and jump. Across all three activities there appears to be good similarity between the pattern of sagittal and frontal joint angles provided by the two systems. However, an offset in the absolute values provided by the two systems is evident, especially for the hip in the sagittal plane (Figure 5) and the knee and ankle in the transverse plane (Figure 7).

**Figure 8:**
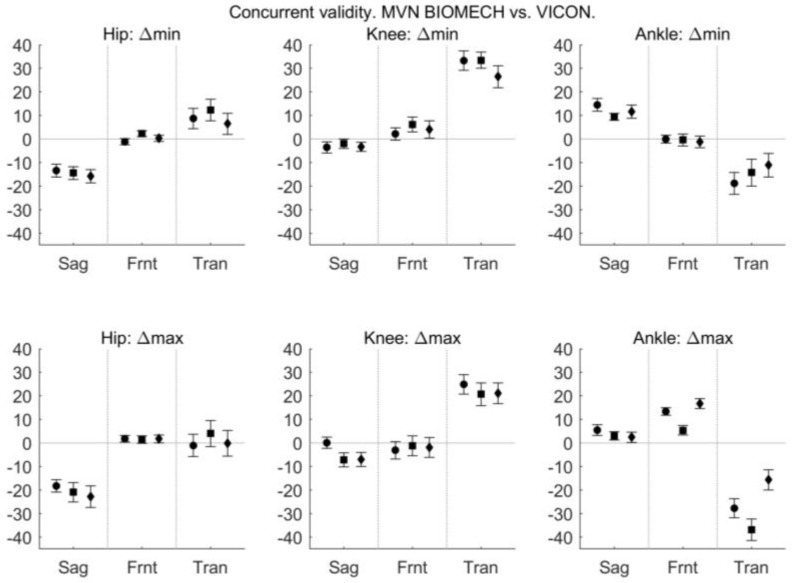

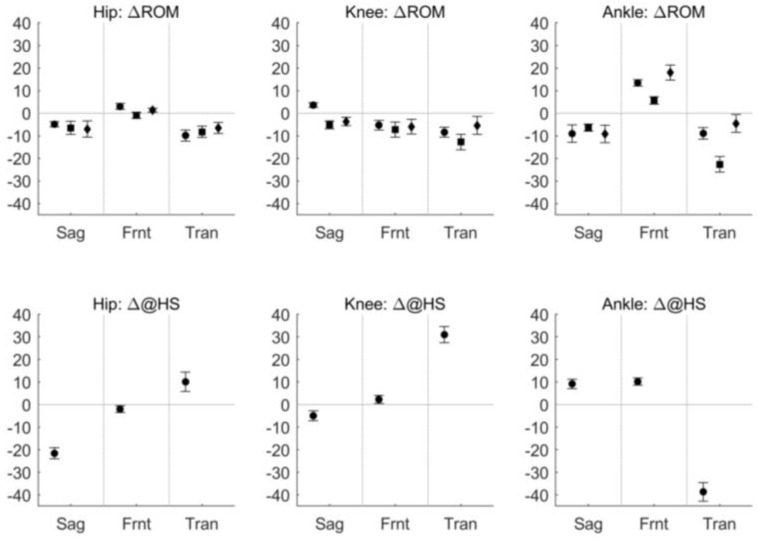
Difference between MVN BIOMECH and VICON data at discrete, clinically relevant events. The difference in minimum angle (Δmin; **top row**), maximum angle (Δmax; **second row**), range of motion (ΔROM; **third row**) and angle at heel strike (Δ@HS; **bottom row**) between the MVN BIOMECH and VICON systems for the hip (**left**), knee (**centre**) and ankle (**right**) joints in the sagittal (Sag), frontal (Frnt) and transverse (Tran) planes of movement. The three data points in each plane correspond to the walk (left-most point; circle), squat (centre point; square) and jump (right-most point; diamond) tasks. Positive values indicate that MVN BIOMECH angle was larger than VICON angle. Error bars indicate 95% confidence interval of the difference.

The difference in joint angle provided by the MVN BIOMECH and VICON systems at discrete, time points is shown in Figure 8.

**Figure 9:**
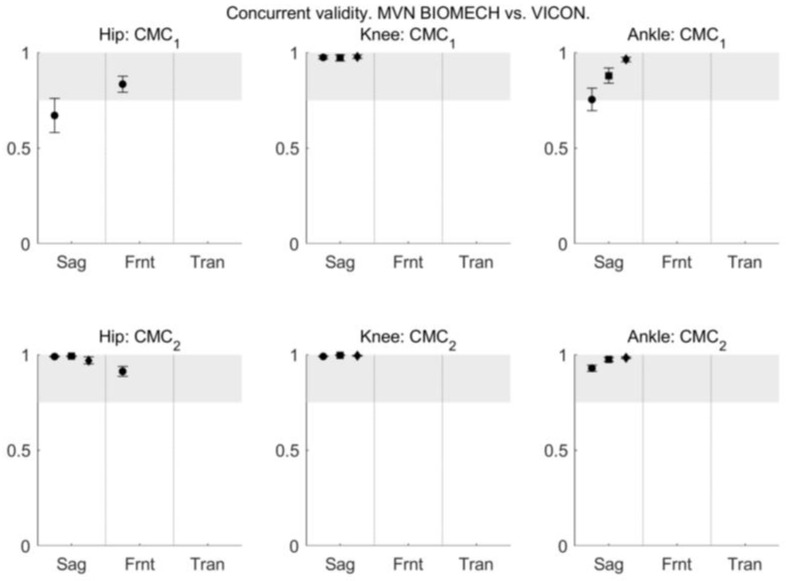
The coefficient of multiple correlation (CMC) between MVN BIOMECH and VICON data before (CMC_1_; **top row**) and after (CMC_2_; **bottom row**) offset removal for the hip (**left**), knee (**centre**) and ankle (**right**) joints in the sagittal (Sag), frontal (Frnt) and transverse (Tran) planes of movement. CMC is reported only for joints and planes where all values are real numbers [39]. Where there are three data points in each plane they correspond to the walk (left-most point; circle), squat (centre point; square) and jump (right-most point; diamond) tasks. Where there is only one data point it corresponds to the walk task. Error bars indicate 95% confidence intervals.

The similarity between the joint angle waveforms obtained from the MVN BIOMECH and VICON systems during walk, squat, and jump was evaluated using CMC and *R*^2^ provided by the LFM. CMC indicated excellent similarity between MVN BIOMECH and VICON systems (CMC_2_ > 0.9) for all three joints in the sagittal plane in all functional activities, and excellent similarity for the hip joint in the frontal plane during walking (Figure 9). CMC is not reported for other planes and activities because of the presence of non-real numbers (see Section 2.6.2).

**Figure 10:**
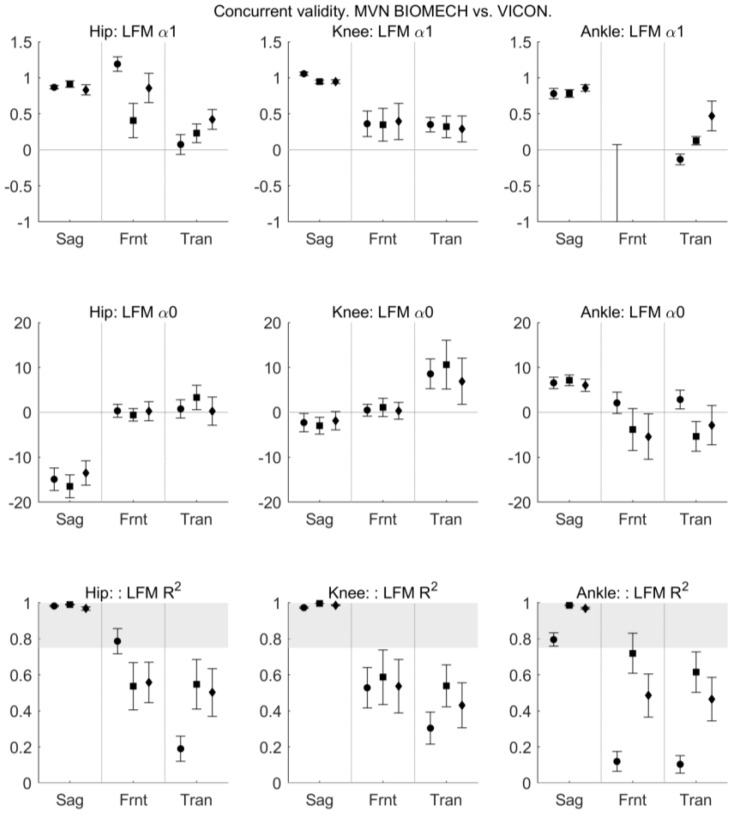
The outcome parameters of linear fit method comparing MVN BIOMECH and VICON data. Outcome parameters are *α*1 (scaling factor; **top row**), *α*0 (scalar addition; **middle row**) and *R*^2^ (strength of the linear relation between the two signals; bottom row) for the hip (**left**), knee (**centre**) and ankle (**right**) joints in the sagittal (Sag), frontal (Frnt) and transverse (Tran) planes of movement. The three data points in each plane correspond to the walk (left-most point; circle), squat (centre point; square) and jump (right-most point; diamond) tasks. Error bars indicate 95% confidence intervals.

*R*^2^ provided by the LFM indicated excellent similarity between MVN BIOMECH and VICON systems (*R*^2^ > 0.8) for sagittal plane angles for all joints across all activities, and fair-to-good similarity (*R*^2^ 0.4–0.8) for transverse and frontal plane angles for all joints during squat and jump and knee joint during walking (Figure 10). The similarity between the two systems was poor for transverse plane hip and ankle joint angles during walking and frontal plane ankle angle during walking.

## 4. Discussion

This study aimed to quantify the within- and between-rater reliability and the concurrent validity of joint angles provided by the commercially available Xsens MVN BIOMECH system during three clinically relevant functional activities. Within-rater reliability was fair-to-excellent in all three planes for hip, knee, and ankle joint angles in all three tasks when comparing the kinematics obtained on two different testing days, i.e., between sessions. Between-rater reliability (between a MSKP and a clinical movement scientist) was fair-to-excellent in all three planes during walking and squatting, and poor-to-high during jumping. Concurrent validity (agreement between the Xsens MVN BIOMECH system and VICON Plug-in Gait, a camera-based motion capture system) was excellent in the sagittal plane for hip, knee, and ankle joint angles in all three tasks and acceptable in frontal and transverse planes in squat and jump. These results indicate that, in the present study, the MVN BIOMECH system was reliably used by an experienced clinician with no prior experience of biomechanical motion capture to quantify lower-limb joint angles in clinically relevant movements. The implications of this for transition of this technology to the clinic are discussed below.

### 4.1. Reliability

For walking, the between- and within-rater reliability of discrete kinematic parameters provided by the MVN BIOMECH system was fair to excellent. The ICC and SEM values are similar to published values for camera-based systems [38,42,43,44,45]. Most reports of camera-based systems have not reported the reliability of ankle angles in the transverse and frontal planes; however, our results for ankle ROM and maximum ankle angle in the transverse plane during walking are comparable to those of Meldrum et al. [46]. According to a systematic review [38], between-rater ICC values for kinematic variables obtained from camera-based systems across joints in sagittal and frontal planes ranged from 0.5 to 0.99. We report ICC values for the MVN BIOMECH system of between 0.65 and 0.99, with a relatively small SEM (<3.0°) for all joints, planes and tasks, with the exception of minimum hip angle in the sagittal plane (ICC 0.39 and SEM 3.0°). This suggests that between- and within-rater reliability of kinematic variables obtained from the MVN BIOMECH system across joints and planes is comparable to or better than those obtained from optoelectronic motion capture systems [38,42,43,44,45,46].

For squatting, the between-rater reliability of kinematic parameters was fair-to-excellent for all parameters across planes. The within-rater reliability was good-to-excellent for all parameters except maximum hip angle in the sagittal plane, where SEM reached 5.5°. For sagittal plane angles, this within-rater reliability compares well to published values for a camera-based system [47]. Between- and within-rater reliability of joint angles in the frontal and transverse planes have not been reported for camera-based systems.

For jumping, the within-rater reliability of kinematic parameters in the sagittal and frontal planes was good-to-excellent, except for maximum hip angle in the sagittal plane, where SEM reached 7°, and the within-rater reliability of kinematic parameters in the transverse plane was fair-to-excellent. The within-rater reliability was slightly higher than that reported for drop jumps captured with a camera-based system [48,49].

**Figure A1:**
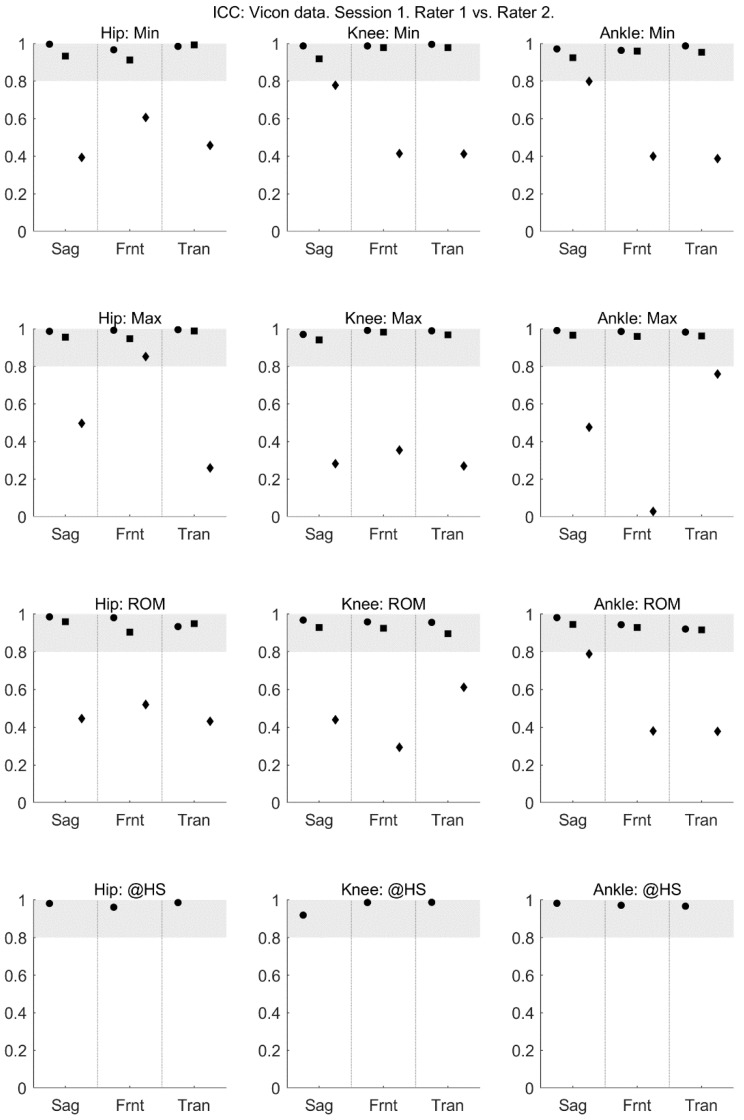
Within-session intraclass correlation coefficient of VICON data collected in session 1. Intraclass correlation coefficient is shown for the minimum angle (min; **top row**), maximum angle (max; **second row**), range of motion (ROM; **third row**) and angle at heel strike (@HS; **bottom row**) for the hip (left-most column), knee (centre column) and ankle (right-most column) joints in the sagittal (Sag), frontal (Frnt) and transverse (Tran) planes of movement. The three data points in each plane correspond to the walk (left-most point; circle), squat (centre point; square) and jump (right-most point; diamond) tasks. Grey shading indicates values considered to indicate excellent reliability.

**Figure A2:**
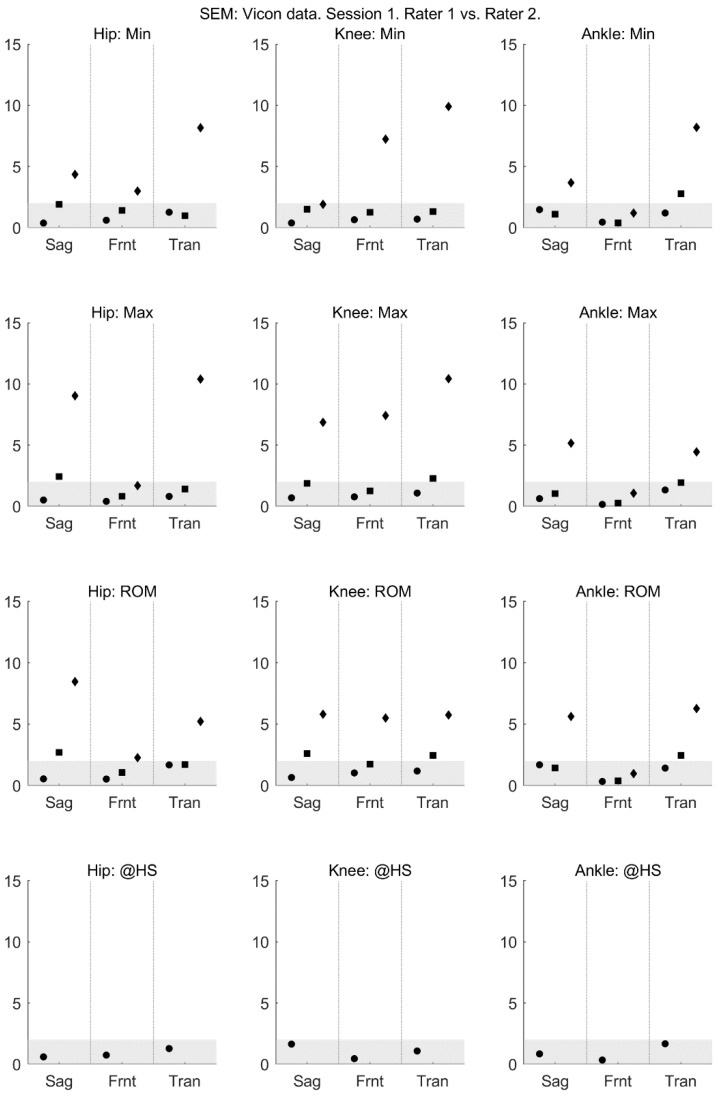
Within-session standard error of measurement of VICON data collected in session 1. Standard error of measurement is shown for the minimum angle (min; **top row**), maximum angle (max; **second row**), range of motion (ROM; **third row**) and angle at heel strike (@HS; **bottom row**) for the hip (left-most column), knee (centre column) and ankle (right-most column) joints in the sagittal (Sag), frontal (Frnt) and transverse (Tran) planes of movement. The three data points in each plane correspond to the walk (left-most point; circle), squat (centre point; square) and jump (right-most point; diamond) tasks. Grey shading indicates values considered to indicate excellent reliability.

For jumping, the between-rater reliability of kinematic parameters was fair for the ankle and hip joints in sagittal, frontal, and transverse planes, and slightly worse for the knee joint. The performance of a dynamic task like jumping may have considerable variability across trials [50], and the poor between-rater reliability across planes and joints observed in the jump might be a true reflection of changes in performance, rather than any deficit of the motion capture system. To help us evaluate whether the poor between-rater reliability was due to the motion capture system or true variability in performance we evaluated the reliability of joint angles obtained from the camera-based motion capture system (VICON) in the first and second half of the session. The markers were not moved between the first and second half of the session; however, ICC was between 0.25 and 0.75 (Appendix B, Figure A1 and Figure A2). This indicates that low between-rater reliability observed with the MVN BIOMECH system is likely due to true variability in performance, and is not a deficit of the motion capture system.

### 4.2. Concurrent Validity

The high CMC_2_ and LFM *R*^2^ values in the sagittal plane indicate that, in this plane of movement, the joint angle waveforms obtained from the MVN BIOMECH system were similar to those obtained from the VICON system. This supports previous work in healthy participants, which also found a high similarity of joint angle waveforms in the sagittal plane between MVN BIOMECH and an Optotrak motion capture system [18,19], and studies that compared kinematic data estimated from Xsens IMUs using custom-developed techniques to an optoelectronic motion capture system [10,13]. Our results extend these previous findings by including a more challenging dynamic task; the vertical jump. The high similarity between MVN BIOMECH and VICON joint angle waveforms in the jump task indicates that the impact that occurs upon initial contact with the ground when landing from the jump did not adversely affect the kinematics provided by the MVN BIOMECH system, and suggests that the MVN BIOMECH system can be used in a dynamic task such as jumping.

Previous studies have reported CMC values for data in frontal and transverse planes [10,18,19], but for the majority of joints and activities we were not able to report CMC in these planes. In line with Ferrari et al. [10], we only report CMC values for joints and planes where all values were real numbers. In the frontal and transverse planes, CMC was a non-real number for at least one participant for most joints and activities. This is likely because of small range of motion in these planes for the activities studied. An alternative measure of similarity between waveforms can be obtained from the LFM *R*^2^ value [41]. There was a good-to-strong relation between frontal plane data from the two systems for the hip during walking and the ankle during squatting, and a moderate relation in frontal and transverse planes for the hip during squatting and jumping, the knee during all activities, and the ankle during jumping. These results are in line with previous reports of a moderate-to-strong relation between waveforms obtained from the MVN BIOMECH system and an optoelectronic system for hip and knee angles during walking [19], stair ascent and descent [19], and manual handling tasks [18]. We found a poor relation (*R*^2^ = 0.1) for the ankle in the transverse and frontal planes during walking, which may be due to the small range of motion in these planes or differences in the anatomical biomechanical definitions between the two systems.

A challenge when validating any new technology is the choice of ‘gold-standard’ against which to validate. This is particularly relevant in movement analysis where there are several biomechanical models available. We chose to validate against the conventional gait model, which is commercially implemented as Plug-in Gait in VICON Nexus software. One limitation of this model is the effect of errors in markers placement on calculated joint angles [51,52,53,54,55]. For example, a small error in the placement of the thigh maker, which is used to define the internal/external rotation of the femur about the line between the hip joint centre, causes appreciable errors in knee joint kinematics, particularly in the frontal and transverse planes (cf. Figure 11.3; [55]). The high values of knee external rotation obtained from Plug-in Gait in this study (see Figure 7) appear non-physiological, and likely indicate errors in thigh marker placement. There are similar potential effects of errors in shank marker placement on the ankle joint kinematics. The validity results, particularly in the frontal and transverse planes, must be interpreted in light of this limitation. The influence of errors in thigh marker placement can be minimised by using a medial epicondyle marker or knee alignment device, and those of errors in the shank marker placement by using a medial malleolus marker, and future studies may consider these techniques. The CMC values for ankle and hip joint angles in frontal and transverse planes during walking in the current study are lower than those reported by Ferrari et al. [10] and Zhang et al. [19]. In addition to the potential influence marker placement errors, this may be impacted by the biomechanical models used by the systems under comparison. Ferrari et al. [10] compared joint angles obtained from IMUs used with a biomechanical model called Outwalk to those obtained from an optoelectronic system (VICON) used with the Calibration Anatomical System Technique [56]. Our comparison for IMU data was VICON Plug-in Gait. Plug-in Gait estimates joint angles based on Kadaba et al. [26], using segment frames that are constructed from anatomical locations identified by optical markers. By contrast, MVN BIOMECH determines segment frames and changes in body posture based on the segment orientations related to an initial neutral pose (the calibration posture), and calculates joint angles directly from the measured segment orientations. The similarity between biomechanical models used by VICON Plug-In-Gait and MVN BIOMECH is lower than that between the two systems used by Ferrari et al. [10], and this may contribute to the lower waveform similarity.

In the current study, there was a difference (offset) in joint angles provided by the MVN BIOMECH and VICON systems that was particularly noticeable for hip flexion/extension and knee and ankle internal/external rotation. This was constant across participants and activities (see Figure 5 and Figure 7 and small confidence intervals in Figure 8), indicating that it is more likely attributable to differences in the biomechanical models, errors in marker placement or deviation of the body position (posture) during calibration from that required by the system than technical issues such as magnetic field. Another possible explanation is that as calibration data for the two systems were collected at different points in the quiet stance trial (see Section 2.4), the posture of the participant may have been slightly different. However, as the data were collected in the same trial, and the participant did not visibly move throughout this trial, we believe that any difference in posture would have been small.

In summary, there was good similarity in joint angle waveforms between the MVN BIOMECH system and VICON Plug-In Gait for all activities, across all joints and planes of motion, with the exception of the ankle joint during walking in the frontal and transverse planes. Despite the similarity in waveforms, the absolute values reported at discrete time points were not similar, particularly at hip and ankle joints. This is because an offset exists between the systems, likely as a result of different biomechanical models employed by the two systems and/or errors in marker placement. This means that the kinematic outputs from the two systems cannot be used interchangeably; for example, hip flexion cannot be compared between one individual who was measured with the MVN BIOMECH system and another individual who was measured with the VICON Plug-in Gait system.

### 4.3. Limitations

Participants in this study were able-bodied adults who attended a single laboratory. Further research is needed to assess the within- and between-rater reliability of the MVN BIOMECH system in pathological populations. The retroreflective markers were placed before the MTw2 trackers, and were not removed until the end of the session (see Section 2.3). This means that the markers were present when both raters placed the MTw2 trackers. The instructions for placement of the MTw2 trackers do not reference anatomical landmarks, therefore we considered the potential impact of the markers being present to be minimal; however, it remains possible that the markers influenced the attachment of the trackers, and thus influenced the inter-rater reliability. We did not use either a medial knee marker or knee alignment device to minimise errors in knee joint angles caused by possible inaccurate thigh marker placement. Our results must be interpreted in with this in mind. Finally, to provide a measure of how repeatable the system is in a clinical environment, participants were not provided with any specific instructions on how to perform the task. This resulted in performance strategy that varied across trials, particularly for the jump. Low reliability in this task is likely influenced by true variations in performance rather than being a reflection of the motion capture system used. This is a clinical strength as the data provide reliability of the system when performance of the task is not constrained.

### 4.4. Clinical Implications and Future Research Directions

Although the reliability and criterion validity of any biomechanical motion analysis system is important, it is not sufficient to recommend transfer of the technology into the clinic. Future work should identify what information is relevant to clinicians, the type of motion capture system that is preferable to clinicians, how the information provided by quantitative motion analysis can best be presented to clinicians, and if such information has any impact on diagnosis or clinical decision making.

## 5. Conclusions

The good between-rater reliability for walk and squat demonstrate that the MVN BIOMECH system provided joint kinematics that were independent of the rater. This is important as the two raters had different backgrounds and expertise: one was a clinically experienced MSKP with no experience of biomechanical motion analysis and the other was an experienced clinical movement scientist. Our results therefore indicate that the data provided by the MVN BIOMECH system are independent of user expertise and the system does not need to be used by an experienced movement scientist. The good within-rater reliability supports the use of this system across multiple participants or sessions. There was excellent similarity between joint angle waveforms obtained from the MVN BIOMECH and VICON systems in the sagittal plane, and acceptable similarity in the frontal and transverse planes in all three tasks. This extends previous reports and indicates that MVN BIOMECH system can be used in a dynamic task such as a jump. However, it must be noted that the MVN BIOMECH and VICON Plug-in Gait systems cannot be used interchangeably. Together, these results indicate that the commercially available MVN BIOMECH system is suited for clinical movement analysis in clinical practice. Future work should evaluate reliability across centres and in pathological populations, and explore the utility of the information that can be provided by this system to clinicians.

## Figures and Tables

**Table 1:** Definition of start and end of movement cycles for each activity.

	Start	End
Walk	Heel strike *	Subsequent heel strike * on the same side
Jump	Local maxima in left knee flexion angle that preceded each local peak in vertical heel position	Local maxima in left knee flexion angle that succeeded each local peak in vertical heel position
Squat	Local minima in left knee flexion angle	Subsequent local minima in left knee flexion angle

* Heel strike was determined as described by Zeni et al. [32], as the local minima in the anterior-posterior position of the heel relative to the sacrum. The sacrum was defined as the average position of left and right posterior superior iliac spines.

## Appendix A

**Table A1:** Within-rater (between-session) reliability of sagittal plane kinematic parameters (left side of the body). Data for each session are mean (standard deviation). Data for the difference are difference [95% confidence interval].

		Walk			Squat			Jump		
		Session 1	Session 2	Difference	Session 1	Session 2	Difference	Session 1	Session 2	Difference
Vicon Hip	Min	−8.8 (6.2)	−9.4 (5.3)	0.6 [−1.0, 2.2]	9.1 (7.3)	8.3 (5.9)	0.8 [−1.0, 2.6]	13.8 (5.6)	14.3 (6.2)	−0.4 [−2.1, 1.2]
	Max	36.6 (4.4)	36.0 (4.1)	0.6 [−0.8, 2.0]	79.9 (11.5)	83.2 (10.8)	−3.3 [−5.7, −0.9]	71.9 (12.7)	74.7 (10.1)	−2.7 [−6.7, 1.3]
	ROM	45.4 (4.5)	45.4 (3.8)	0.0 [−0.9, 0.9]	70.8 (13.7)	74.9 (11.9)	−4.1 [−6.7, −1.5]	58.1 (11.4)	60.4 (9.8)	−2.3 [−6.4, 1.8]
	@HS	35.6 (4.4)	35.0 (4.5)	0.6 [−0.8, 2.1]	-	-	-	-	-	-
Vicon Knee	Min	4.2 (3.4)	2.8 (3.6)	1.4 [−0.0, 2.8]	2.2 (5.3)	0.1 (3.8)	2.1 [0.6, 3.6]	5.8 (4.1)	4.7 (3.9)	1.2 [−0.7, 3.1]
	Max	64.5 (4.0)	62.9 (4.4)	1.6 [−0.3, 3.6]	77.7 (7.7)	79.2 (9.0)	−1.5 [−4.3, 1.3]	80.5 (8.1)	80.8 (5.8)	−0.3 [−3.1, 2.5]
	ROM	60.4 (3.7)	60.1 (3.4)	0.3 [−0.8, 1.3]	75.6 (9.7)	79.2 (9.0)	−3.6 [−6.9, −0.4]	74.7 (7.7)	76.1 (6.2)	−1.4 [−3.8, 0.9]
	@HS	8.5 (5.8)	7.2 (6.0)	1.2 [−1.1, 3.5]	-	-	-	-	-	-
Vicon Ankle	Min	−32.3 (8.7)	−33.3 (13.7)	1.0 [−4.4, 6.3]	−7.1 (4.0)	−10.4 (7.3)	3.3 [0.5, 6.1]	−51.9 (8.2)	−52.1 (15.3)	0.2 [−5.1, 5.4]
	Max	12.3 (6.5)	12.6 (9.9)	−0.3 [−3.2, 2.5]	21.5 (5.6)	19.6 (6.7)	1.8 [−0.3, 3.9]	27.7 (7.1)	27.4 (9.1)	0.3 [−2.0, 2.6]
	ROM	44.7 (12.4)	45.9 (22.6)	−1.3 [−8.6, 6.0]	28.6 (6.1)	30.1 (10.9)	−1.5 [−5.4, 2.5]	79.6 (12.2)	79.5 (22.6)	0.1 [−6.9, 7.2]
	@HS	−9.1 (6.4)	−10.4 (8.0)	1.3 [−1.3, 3.9]	-	-	-	-	-	-
Xsens Hip	Min	−21.9 (3.8)	−21.4 (3.7)	−0.6 [−2.0, 0.9]	−5.1 (5.2)	−2.5 (7.0)	−2.6 [−5.4, 0.2]	−1.9 (6.0)	1.3 (7.3)	−3.2 [−5.4, −1.1]
	Max	18.6 (5.8)	18.7 (6.1)	−0.2 [−2.2, 1.8]	58.9 (18.0)	65.9 (15.6)	−6.9 [−11.2, −2.7]	49.0 (19.9)	54.1 (17.0)	−5.1 [−10.4, 0.2]
	ROM	40.5 (4.5)	40.1 (4.8)	0.4 [−0.7, 1.4]	64.0 (16.8)	68.4 (15.2)	−4.3 [−7.3, −1.4]	50.9 (15.9)	52.7 (14.0)	−1.9 [−6.8, 3.1]
	@HS	14.3 (5.0)	14.9 (5.6)	−0.7 [−2.6, 1.3]	-	-	-	-	-	-
Xsens Knee	Min	0.9 (4.1)	1.4 (4.2)	−0.5 [−1.6, 0.7]	0.4 (3.4)	0.2 (2.6)	0.2 [−0.9, 1.2]	2.6 (3.1)	3.5 (3.2)	−0.9 [−2.0, 0.2]
	Max	64.8 (5.3)	65.1 (5.6)	−0.3 [−1.6, 1.0]	70.6 (10.3)	74.4 (9.3)	−3.8 [−6.7, −0.8]	73.6 (10.6)	75.8 (9.6)	−2.2 [−4.9, 0.6]
	ROM	63.9 (2.7)	63.7 (3.3)	0.2 [−0.7, 1.0]	70.3 (10.7)	74.2 (9.8)	−4.0 [−7.0, −0.9]	71.0 (9.7)	72.2 (8.3)	−1.3 [−3.7, 1.1]
	@HS	3.7 (6.1)	4.2 (5.6)	−0.5 [−2.5, 1.4]	-	-	-	-	-	-
Xsens Ankle	Min	−17.7 (5.0)	−17.1 (4.9)	−0.6 [−2.3, 1.1]	2.5 (2.2)	1.9 (2.1)	0.6 [−0.3, 1.4]	−40.0 (5.4)	−39.1 (5.5)	−1.0 [−2.7, 0.8]
	Max	17.8 (2.5)	18.2 (2.6)	−0.4 [−1.0, 0.2]	24.5 (5.4)	24.4 (5.4)	0.1 [−1.2, 1.5]	30.2 (6.3)	30.7 (7.0)	−0.5 [−2.1, 1.1]
	ROM	35.6 (5.7)	35.4 (5.4)	0.2 [−1.6, 2.0]	22.0 (5.5)	22.5 (5.5)	−0.4 [−2.1, 1.3]	70.2 (9.3)	69.7 (9.8)	0.5 [−1.3, 2.3]
	@HS	0.1 (3.2)	0.1 (3.8)	0.0 [−0.9, 0.9]	-	-	-	-	-	-

**Table A2:** Within-rater (between-session) reliability of frontal plane kinematic parameters (left side of the body).

		Walk			Squat			Jump		
		Session 1	Session 2	Difference	Session 1	Session 2	Difference	Session 1	Session 2	Difference
Vicon Hip	Min	−8.2 (3.3)	−8.4 (3.4)	0.2 [−1.1, 1.5]	−8.0 (4.8)	−8.7 (4.6)	0.7 [−1.1, 2.5]	−9.2 (4.8)	−9.1 (4.8)	−0.1 [−1.8, 1.6]
	Max	7.4 (4.6)	7.3 (3.6)	0.0 [−1.3, 1.4]	−1.6 (3.6)	−1.9 (2.8)	0.3 [−1.1, 1.7]	−2.2 (4.4)	−2.4 (3.6)	0.3 [−1.3, 1.8]
	ROM	15.6 (3.9)	15.7 (3.7)	−0.2 [−0.7, 0.4]	6.4 (3.5)	6.8 (3.7)	−0.4 [−1.5, 0.8]	7.0 (3.3)	6.6 (3.3)	0.4 [−0.6, 1.3]
	@HS	−0.4 (3.8)	−0.6 (3.0)	0.2 [−1.4, 1.7]	-	-		-	-	
Vicon Knee	Min	−6.5 (5.7)	−5.8 (7.8)	−0.7 [−3.3, 2.0]	−8.7 (8.3)	−6.2 (8.0)	−2.4 [−5.6, 0.7]	−8.3 (9.5)	−6.3 (9.8)	−2.1 [−5.7, 1.6]
	Max	8.9 (8.4)	11.0 (12.0)	−2.1 [−6.4, 2.2]	7.0 (9.3)	9.4 (13.1)	−2.4 [−6.7, 1.8]	6.7 (9.2)	9.9 (13.7)	−3.1 [−7.4, 1.1]
	ROM	15.4 (5.1)	16.8 (7.9)	−1.4 [−4.3, 1.5]	15.6 (6.4)	15.6 (9.8)	0.0 [−4.0, 4.1]	15.1 (6.5)	16.2 (9.2)	−1.1 [−5.0, 2.8]
	@HS	−3.1 (4.0)	−2.0 (4.4)	−1.1 [−2.4, 0.2]	-	-		-	-	
Vicon Ankle	Min	−3.6 (2.3)	−3.9 (3.8)	0.3 [−1.2, 1.8]	−3.2 (1.9)	−3.4 (3.2)	0.2 [−1.2, 1.5]	−2.4 (1.5)	−2.5 (2.7)	0.1 [−1.0, 1.2]
	Max	−0.9 (1.3)	−0.7 (1.6)	−0.3 [−1.0, 0.4]	−0.8 (1.3)	−0.6 (1.5)	−0.3 [−0.9, 0.3]	−0.6 (1.1)	−0.2 (1.4)	−0.4 [−1.0, 0.2]
	ROM	2.6 (1.4)	3.2 (2.6)	−0.6 [−1.6, 0.4]	2.4 (1.4)	2.9 (2.3)	−0.4 [−1.4, 0.6]	1.8 (1.2)	2.3 (2.0)	−0.5 [−1.4, 0.4]
	@HS	−2.8 (2.1)	−3.0 (2.9)	0.2 [−1.0, 1.4]	-	-		-	-	
Xsens Hip	Min	−9.3 (3.0)	−9.7 (2.6)	0.3 [−0.6, 1.3]	−5.5 (3.5)	−6.3 (3.9)	0.8 [−0.6, 2.3]	−8.7 (4.1)	−9.6 (4.4)	0.9 [−0.1, 1.9]
	Max	9.2 (2.7)	9.1 (2.4)	0.1 [−0.5, 0.8]	0.0 (3.6)	−0.5 (3.0)	0.6 [−0.8, 1.9]	−0.3 (4.1)	−1.0 (4.0)	0.7 [−0.5, 1.9]
	ROM	18.6 (3.8)	18.8 (3.0)	−0.2 [−1.2, 0.8]	5.5 (2.3)	5.7 (2.3)	−0.3 [−1.3, 0.8]	8.5 (2.7)	8.6 (2.6)	−0.2 [−1.2, 0.9]
	@HS	−2.3 (3.5)	−2.9 (3.5)	0.6 [−1.1, 2.3]	-	-		-	-	
Xsens Knee	Min	−4.6 (3.2)	−4.2 (3.5)	−0.3 [−1.7, 1.1]	−2.9 (2.8)	−3.5 (2.8)	0.6 [−0.5, 1.6]	−4.8 (4.7)	−4.6 (4.2)	−0.1 [−1.6, 1.3]
	Max	5.2 (5.6)	5.8 (5.0)	−0.6 [−1.9, 0.8]	5.3 (5.9)	4.8 (5.6)	0.5 [−1.2, 2.2]	4.3 (5.6)	4.6 (5.5)	−0.3 [−1.6, 1.0]
	ROM	9.8 (4.1)	10.0 (3.8)	−0.2 [−1.6, 1.1]	8.2 (4.5)	8.2 (4.4)	−0.1 [−1.2, 1.1]	9.0 (3.8)	9.2 (4.6)	−0.2 [−1.3, 1.0]
	@HS	−1.0 (1.4)	−0.5 (1.9)	−0.5 [−1.4, 0.4]	-	-		-	-	
Xsens Ankle	Min	−3.6 (2.9)	−3.6 (2.7)	−0.1 [−1.3, 1.2]	−3.6 (5.3)	−2.1 (5.9)	−1.4 [−3.2, 0.4]	−3.7 (5.0)	−4.0 (4.3)	0.3 [−1.2, 1.7]
	Max	12.4 (3.7)	13.2 (4.7)	−0.8 [−3.1, 1.5]	4.6 (4.1)	5.9 (4.2)	−1.3 [−2.7, 0.1]	15.8 (5.2)	14.8 (5.0)	1.1 [−1.1, 3.2]
	ROM	16.0 (3.1)	16.8 (4.6)	−0.8 [−2.8, 1.3]	8.1 (3.8)	8.0 (3.7)	0.1 [−1.0, 1.3]	19.5 (7.7)	18.8 (7.5)	0.8 [−2.4, 4.0]
	@HS	7.3 (3.3)	7.1 (3.4)	0.2 [−1.5, 2.0]	-	-		-	-	

Data for each session are mean (standard deviation). Data for the difference are difference [95% confidence interval].

**Table A3:** Within-rater (between-session) reliability of transverse plane kinematic parameters (left side of the body).

		Walk			Squat			Jump		
		Session 1	Session 2	Difference	Session 1	Session 2	Difference	Session 1	Session 2	Difference
Vicon Hip	Min	−14.8 (10.2)	−10.4 (12.4)	−4.4 [−9.2, 0.3]	−14.9 (11.2)	−9.2 (13.2)	−5.6 [−10.6, −0.7]	−15.7 (11.1)	−11.2 (14.5)	−4.5 [−9.7, 0.6]
	Max	7.4 (12.0)	9.7 (15.3)	−2.2 [−8.9, 4.4]	1.5 (13.0)	5.2 (13.5)	−3.7 [−9.9, 2.5]	2.1 (12.1)	5.1 (15.7)	−3.0 [−9.1, 3.1]
	ROM	22.2 (6.6)	20.0 (7.3)	2.2 [−1.7, 6.1]	16.4 (7.7)	14.5 (4.8)	1.9 [−0.8, 4.7]	17.8 (6.9)	16.2 (6.5)	1.6 [−0.9, 4.0]
	@HS	−12.1 (11.0)	−7.2 (13.7)	−5.0 [−10.2, 0.3]	-	-		-	-	
Vicon Knee	Min	−44.5 (10.5)	−46.8 (11.4)	2.3 [−2.7, 7.4]	−35.8 (8.9)	−40.0 (9.2)	4.2 [0.3, 8.2]	−34.2 (12.9)	−35.0 (11.5)	0.8 [−4.5, 6.1]
	Max	−22.0 (10.4)	−20.4 (12.9)	−1.6 [−6.1, 2.9]	−15.6 (12.6)	−12.3 (14.4)	−3.3 [−8.3, 1.8]	−16.0 (12.2)	−13.8 (17.0)	−2.2 [−8.2, 3.8]
	ROM	22.5 (5.7)	26.4 (8.4)	−3.9 [−6.8, −1.0]	20.2 (7.6)	27.7 (14.0)	−7.5 [−12.0, −3.0]	18.3 (9.2)	21.2 (12.3)	−3.0 [−7.3, 1.4]
	@HS	−35.8 (9.8)	−38.8 (9.8)	3.0 [−0.8, 6.8]	-	-		-	-	
Vicon Ankle	Min	12.6 (10.5)	8.1 (15.2)	4.5 [−0.7, 9.7]	9.5 (12.9)	6.6 (16.2)	2.9 [−2.5, 8.2]	6.1 (10.5)	2.0 (14.7)	4.2 [−0.6, 8.9]
	Max	40.9 (10.1)	39.0 (14.0)	1.8 [−2.9, 6.6]	37.2 (9.8)	35.7 (13.7)	1.5 [−3.3, 6.3]	28.9 (9.1)	26.6 (13.5)	2.3 [−2.3, 6.8]
	ROM	28.3 (5.1)	30.9 (6.4)	−2.6 [−5.2, −0.1]	27.7 (8.5)	29.1 (10.1)	−1.4 [−4.8, 2.1]	22.8 (7.9)	24.7 (9.4)	−1.9 [−4.3, 0.5]
	@HS	35.3 (9.3)	33.1 (14.2)	2.2 [−2.2, 6.7]	-	-		-	-	
Xsens Hip	Min	−6.7 (3.9)	−6.8 (4.2)	0.2 [−1.5, 1.9]	−2.9 (3.7)	−5.0 (4.8)	2.1 [−0.0, 4.2]	−9.8 (4.4)	−10.5 (4.3)	0.7 [−1.1, 2.6]
	Max	5.7 (4.5)	5.4 (4.5)	0.3 [−1.2, 1.9]	5.0 (4.6)	3.5 (5.3)	1.5 [−0.4, 3.5]	1.3 (5.2)	0.9 (4.8)	0.4 [−1.3, 2.0]
	ROM	12.4 (2.7)	12.2 (3.6)	0.2 [−1.2, 1.5]	7.9 (3.3)	8.5 (3.0)	−0.6 [−1.9, 0.7]	11.0 (3.6)	11.4 (4.0)	−0.4 [−1.4, 0.7]
	@HS	−2.7 (4.6)	−2.0 (5.2)	−0.7 [−2.4, 0.9]	-	-		-	-	
Xsens Knee	Min	−11.1 (4.8)	−12.1 (3.8)	1.0 [−0.6, 2.5]	−2.3 (3.8)	−3.1 (2.8)	0.8 [−0.7, 2.3]	−7.4 (4.7)	−8.2 (4.7)	0.7 [−0.8, 2.2]
	Max	3.0 (4.0)	1.9 (2.7)	1.1 [−0.7, 2.8]	4.8 (4.8)	4.4 (3.4)	0.4 [−1.8, 2.7]	5.0 (4.2)	4.8 (4.3)	0.2 [−1.7, 2.1]
	ROM	14.2 (3.8)	14.0 (3.4)	0.1 [−0.9, 1.2]	7.1 (2.6)	7.5 (2.4)	−0.4 [−1.6, 0.9]	12.5 (3.2)	13.0 (4.1)	−0.5 [−1.8, 0.8]
	@HS	−4.7 (5.8)	−6.0 (5.1)	1.3 [−0.6, 3.2]	-	-		-	-	
Xsens Ankle	Min	−6.4 (5.0)	−6.7 (3.7)	0.3 [−2.2, 2.9]	−4.9 (5.7)	−5.4 (5.5)	0.5 [−2.1, 3.1]	−5.1 (4.1)	−4.2 (6.2)	−0.9 [−3.6, 1.9]
	Max	13.1 (5.4)	14.1 (5.4)	−0.9 [−3.3, 1.5]	0.4 (5.7)	−0.0 (5.2)	0.4 [−2.2, 3.0]	13.3 (4.2)	13.2 (6.2)	0.2 [−2.5, 2.8]
	ROM	19.5 (4.0)	20.8 (4.5)	−1.2 [−3.2, 0.7]	5.3 (1.6)	5.4 (2.1)	−0.1 [−0.9, 0.7]	18.4 (3.6)	17.4 (3.8)	1.0 [−0.9, 3.0]
	@HS	−3.0 (4.3)	−3.0 (2.8)	−0.0 [−2.2, 2.1]	-	-		-	-	

Data for each session are mean (standard deviation). Data for the difference are difference [95% confidence interval].

**Table A4:** Between-rater (within-session) reliability of sagittal plane kinematic parameters (left side of the body).

		Walk			Squat			Jump		
		Session 1	Session 2	Difference	Session 1	Session 2	Difference	Session 1	Session 2	Difference
Vicon Hip	Min	−8.7 (6.1)	−8.5 (6.3)	−0.2 [−0.5, 0.1]	9.4 (7.4)	8.9 (6.9)	0.5 [−0.9, 2.0]	13.8 (5.5)	13.8 (5.7)	−0.0 [−0.9, 0.8]
	Max	36.7 (4.3)	36.6 (4.7)	0.1 [−0.3, 0.5]	80.2 (11.4)	80.6 (10.8)	−0.3 [−2.2, 1.6]	72.8 (13.2)	72.2 (12.9)	0.7 [−1.6, 2.9]
	ROM	45.4 (4.4)	45.1 (4.5)	0.3 [−0.1, 0.8]	70.8 (13.4)	71.7 (13.1)	−0.9 [−3.0, 1.3]	59.0 (12.0)	58.3 (12.4)	0.7 [−1.4, 2.9]
	@HS	35.7 (4.4)	35.9 (4.8)	−0.2 [−0.7, 0.4]	-	-		-	-	
Vicon Knee	Min	4.1 (3.3)	3.9 (3.5)	0.2 [−0.1, 0.5]	2.3 (5.3)	1.7 (4.2)	0.6 [−0.5, 1.7]	5.6 (4.1)	6.0 (4.8)	−0.4 [−1.7, 0.9]
	Max	64.6 (3.9)	64.3 (4.0)	0.3 [−0.3, 0.8]	78.0 (7.7)	77.6 (7.8)	0.4 [−1.1, 1.8]	81.3 (8.9)	80.0 (8.3)	1.3 [−0.6, 3.3]
	ROM	60.4 (3.6)	60.4 (3.7)	0.1 [−0.5, 0.6]	75.7 (9.5)	75.9 (8.6)	−0.3 [−2.2, 1.7]	75.7 (8.9)	73.9 (8.0)	1.7 [−0.1, 3.6]
	@HS	8.3 (5.7)	8.3 (5.8)	0.1 [−1.2, 1.4]	-	-		-	-	
Vicon Ankle	Min	−32.3 (8.5)	−32.0 (8.6)	−0.3 [−1.5, 0.9]	−7.1 (3.9)	−7.6 (4.3)	0.5 [−0.4, 1.4]	−51.9 (8.0)	−50.6 (9.4)	−1.4 [−3.6, 0.9]
	Max	12.2 (6.5)	12.2 (6.3)	−0.1 [−0.6, 0.5]	21.3 (5.6)	21.1 (5.9)	0.3 [−0.6, 1.1]	27.3 (7.2)	28.0 (7.1)	−0.7 [−1.6, 0.1]
	ROM	44.4 (12.2)	44.2 (11.7)	0.2 [−1.1, 1.5]	28.5 (6.0)	28.7 (6.3)	−0.2 [−1.4, 1.0]	79.2 (12.1)	78.6 (13.3)	0.6 [−1.2, 2.5]
	@HS	−9.2 (6.3)	−9.4 (6.7)	0.2 [−0.5, 1.0]	-	-		-	-	
Xsens Hip	Min	−22.1 (3.8)	−20.8 (3.4)	−1.2 [−3.1, 0.6]	−5.0 (5.1)	−4.6 (5.1)	−0.4 [−2.3, 1.6]	−2.0 (5.9)	−0.8 (6.0)	−1.2 [−3.2, 0.8]
	Max	18.5 (5.7)	19.3 (4.7)	−0.8 [−2.8, 1.1]	59.3 (17.8)	61.5 (14.5)	−2.2 [−5.6, 1.1]	50.0 (20.1)	51.3 (17.5)	−1.3 [−5.5, 3.0]
	ROM	40.5 (4.5)	40.1 (5.2)	0.4 [−0.4, 1.3]	64.3 (16.5)	66.2 (16.3)	−1.8 [−4.5, 0.8]	52.1 (16.6)	52.1 (15.7)	−0.1 [−3.1, 2.9]
	@HS	14.1 (5.0)	15.1 (3.9)	−0.9 [−2.8, 1.0]	-	-		-	-	
Xsens Knee	Min	0.6 (4.2)	0.9 (3.7)	−0.3 [−1.3, 0.7]	0.4 (3.3)	0.5 (3.7)	−0.1 [−1.2, 0.9]	2.3 (3.5)	2.8 (3.9)	−0.5 [−1.9, 0.8]
	Max	64.7 (5.2)	64.6 (5.1)	0.1 [−1.4, 1.6]	70.9 (10.1)	71.3 (9.9)	−0.4 [−2.4, 1.7]	74.3 (10.9)	72.8 (10.1)	1.5 [−1.4, 4.3]
	ROM	64.0 (2.8)	63.7 (3.6)	0.3 [−0.8, 1.4]	70.5 (10.5)	70.7 (10.5)	−0.2 [−2.1, 1.6]	72.0 (10.8)	70.0 (10.0)	2.0 [−0.8, 4.8]
	@HS	3.4 (6.2)	3.6 (5.2)	−0.2 [−1.8, 1.4]	-	-		-	-	
Xsens Ankle	Min	−17.8 (4.9)	−20.3 (5.2)	2.5 [0.9, 4.1]	2.3 (2.3)	1.7 (2.8)	0.6 [−0.3, 1.4]	−40.3 (5.5)	−41.6 (6.6)	1.3 [−0.6, 3.1]
	Max	17.6 (2.6)	17.3 (3.0)	0.4 [−0.4, 1.1]	24.4 (5.3)	22.7 (5.9)	1.7 [0.6, 2.7]	29.8 (6.4)	29.3 (6.7)	0.5 [−0.7, 1.7]
	ROM	35.4 (5.6)	37.6 (5.5)	−2.2 [−3.6, −0.7]	22.1 (5.4)	21.0 (5.5)	1.1 [0.1, 2.1]	70.1 (9.1)	70.9 (10.8)	−0.8 [−2.9, 1.3]
	@HS	0.0 (3.2)	−2.4 (3.5)	2.4 [1.4, 3.5]	-	-		-	-	

Data for each session are mean (standard deviation). Data for the difference are difference [95% confidence interval].

**Table A5:** Between-rater (within-session) reliability of frontal plane kinematic parameters (left side of the body).

		Walk			Squat			Jump		
		Session 1	Session 2	Difference	Session 1	Session 2	Difference	Session 1	Session 2	Difference
Vicon Hip	Min	−8.1 (3.3)	−8.0 (3.4)	−0.0 [−0.5, 0.5]	−8.1 (4.7)	−9.2 (5.3)	1.1 [−0.1, 2.2]	−9.4 (4.7)	−10.0 (4.3)	0.6 [−0.1, 1.3]
	Max	7.4 (4.5)	7.5 (4.4)	−0.1 [−0.4, 0.2]	−1.6 (3.6)	−1.9 (3.4)	0.3 [−0.3, 1.0]	−2.1 (4.3)	−2.0 (4.0)	−0.1 [−0.7, 0.6]
	ROM	15.5 (3.8)	15.5 (3.7)	−0.1 [−0.5, 0.4]	6.5 (3.4)	7.2 (3.7)	−0.7 [−1.6, 0.1]	7.3 (3.5)	8.0 (3.7)	−0.7 [−1.6, 0.2]
	@HS	−0.4 (3.7)	−0.7 (3.6)	0.3 [−0.3, 0.9]	-	-		-	-	
Vicon Knee	Min	−6.7 (5.6)	−6.5 (5.4)	−0.2 [−0.7, 0.3]	−8.9 (8.2)	−8.6 (8.6)	−0.3 [−1.4, 0.7]	−8.6 (9.4)	−8.3 (8.8)	−0.3 [−1.4, 0.7]
	Max	8.4 (8.6)	7.7 (8.7)	0.6 [0.0, 1.3]	6.4 (9.5)	6.3 (9.8)	0.1 [−1.0, 1.1]	6.3 (9.3)	6.3 (9.3)	0.0 [−1.0, 1.0]
	ROM	15.1 (5.2)	14.2 (5.1)	0.8 [−0.0, 1.6]	15.3 (6.5)	14.9 (7.4)	0.4 [−1.1, 1.9]	14.9 (6.4)	14.6 (6.3)	0.3 [−0.7, 1.4]
	@HS	−3.3 (3.9)	−3.0 (3.8)	−0.3 [−0.7, 0.1]	-	-		-	-	
Vicon Ankle	Min	−3.6 (2.3)	−3.6 (2.5)	0.1 [−0.3, 0.5]	−3.2 (1.9)	−3.3 (2.0)	0.1 [−0.2, 0.4]	−2.4 (1.5)	−2.6 (1.8)	0.2 [−0.2, 0.7]
	Max	−0.9 (1.3)	−1.0 (1.4)	0.0 [−0.1, 0.2]	−0.8 (1.3)	−1.0 (1.4)	0.2 [−0.1, 0.4]	−0.6 (1.1)	−0.9 (1.1)	0.3 [−0.2, 0.9]
	ROM	2.6 (1.4)	2.7 (1.5)	−0.0 [−0.3, 0.2]	2.4 (1.4)	2.3 (1.2)	0.1 [−0.2, 0.4]	1.8 (1.2)	1.7 (1.0)	0.1 [−0.2, 0.4]
	@HS	−2.8 (2.0)	−2.9 (2.0)	0.0 [−0.2, 0.3]	-	-		-	-	
Xsens Hip	Min	−9.2 (3.0)	−9.6 (3.6)	0.4 [−0.5, 1.3]	−5.7 (3.6)	−7.0 (4.6)	1.2 [0.1, 2.4]	−9.0 (4.3)	−9.7 (4.3)	0.6 [−0.4, 1.6]
	Max	9.2 (2.7)	9.0 (3.0)	0.2 [−0.5, 1.0]	−0.2 (3.6)	−0.7 (4.1)	0.6 [−0.7, 1.8]	−0.3 (4.0)	−0.8 (4.1)	0.5 [−0.7, 1.7]
	ROM	18.4 (3.8)	18.6 (3.5)	−0.1 [−1.1, 0.8]	5.6 (2.3)	6.3 (2.5)	−0.7 [−1.5, 0.1]	8.7 (2.9)	8.8 (2.8)	−0.1 [−0.8, 0.5]
	@HS	−2.4 (3.4)	−3.8 (3.7)	1.4 [0.2, 2.6]	-	-		-	-	
Xsens Knee	Min	−4.5 (3.2)	−3.0 (2.6)	−1.4 [−2.3, −0.6]	−2.8 (2.8)	−1.9 (2.1)	−0.9 [−1.7, −0.1]	−4.6 (4.7)	−3.3 (3.5)	−1.3 [−2.4, −0.2]
	Max	5.3 (5.5)	7.3 (5.4)	−1.9 [−3.0, −0.8]	5.3 (5.8)	5.8 (4.3)	−0.5 [−2.1, 1.0]	4.4 (5.5)	5.3 (3.9)	−0.9 [−2.2, 0.4]
	ROM	9.8 (4.1)	10.3 (4.4)	−0.5 [−1.6, 0.6]	8.1 (4.4)	7.7 (3.6)	0.3 [−1.2, 1.9]	9.0 (3.7)	8.6 (2.3)	0.4 [−0.9, 1.7]
	@HS	−0.9 (1.4)	0.3 (1.4)	−1.2 [−1.8, −0.6]	-	-		-	-	
Xsens Ankle	Min	−3.6 (2.9)	−5.0 (3.1)	1.4 [0.4, 2.4]	−3.6 (5.2)	−3.5 (5.6)	−0.0 [−1.4, 1.3]	−3.6 (4.9)	−4.2 (4.9)	0.6 [−0.8, 2.1]
	Max	12.5 (3.7)	12.5 (4.1)	−0.0 [−1.6, 1.5]	4.6 (4.0)	4.6 (4.2)	−0.0 [−1.3, 1.3]	16.2 (5.4)	16.2 (5.8)	0.0 [−2.2, 2.2]
	ROM	16.0 (3.0)	17.4 (3.8)	−1.4 [−2.7, −0.1]	8.1 (3.7)	8.1 (3.4)	0.0 [−0.8, 0.9]	19.8 (7.6)	20.4 (6.8)	−0.6 [−3.5, 2.3]
	@HS	7.3 (3.3)	7.1 (3.3)	0.2 [−1.2, 1.7]	-	-		-	-	

Data for each session are mean (standard deviation). Data for the difference are difference [95% confidence interval].

**Table A6:** Between-rater (within-session) reliability of transverse plane kinematic parameters (left side of the body).

		Walk			Squat			Jump		
		Session 1	Session 2	Difference	Session 1	Session 2	Difference	Session 1	Session 2	Difference
Vicon Hip	Min	−15.2 (10.2)	−14.9 (10.2)	−0.3 [−1.4, 0.7]	−15.2 (11.1)	−15.4 (10.8)	0.2 [−0.6, 1.0]	−16.1 (11.0)	−17.2 (10.7)	1.2 [−0.4, 2.7]
	Max	7.0 (12.0)	5.9 (12.0)	1.0 [0.3, 1.7]	1.0 (13.0)	0.7 (13.4)	0.3 [−0.9, 1.4]	1.6 (12.0)	1.2 (11.9)	0.5 [−0.7, 1.6]
	ROM	22.1 (6.4)	20.8 (5.9)	1.3 [0.1, 2.6]	16.1 (7.6)	16.0 (8.3)	0.1 [−1.3, 1.5]	17.7 (6.8)	18.4 (9.1)	−0.7 [−2.4, 1.0]
	@HS	−12.6 (11.0)	−12.5 (11.0)	−0.1 [−1.1, 1.0]	-	-		-	-	
Vicon Knee	Min	−44.2 (10.3)	−43.9 (10.1)	−0.3 [−0.9, 0.3]	−35.5 (8.8)	−36.5 (8.6)	1.0 [−0.0, 2.1]	−33.9 (12.8)	−32.0 (15.1)	−1.9 [−6.5, 2.8]
	Max	−21.7 (10.3)	−22.0 (10.7)	0.3 [−0.6, 1.2]	−15.6 (12.3)	−16.2 (11.6)	0.7 [−1.1, 2.4]	−15.8 (12.0)	−14.7 (14.3)	−1.1 [−3.3, 1.2]
	ROM	22.5 (5.5)	21.9 (6.2)	0.6 [−0.4, 1.6]	20.0 (7.5)	20.3 (8.9)	−0.4 [−2.4, 1.7]	18.1 (9.1)	17.3 (6.7)	0.8 [−1.7, 3.4]
	@HS	−35.5 (9.8)	−35.9 (9.4)	0.4 [−0.4, 1.3]	-	-		-	-	
Vicon Ankle	Min	12.6 (10.3)	13.2 (10.4)	−0.6 [−1.6, 0.4]	9.5 (12.6)	10.9 (12.8)	−1.4 [−3.6, 0.8]	6.2 (10.3)	5.3 (13.9)	0.8 [−1.6, 3.3]
	Max	41.1 (9.9)	41.1 (10.0)	−0.0 [−1.1, 1.0]	37.3 (9.7)	37.8 (10.0)	−0.5 [−2.1, 1.1]	29.2 (9.0)	27.2 (15.3)	2.0 [−3.1, 7.0]
	ROM	28.5 (5.0)	27.9 (4.9)	0.6 [−0.5, 1.7]	27.8 (8.3)	27.0 (8.3)	0.9 [−1.1, 2.8]	23.0 (7.8)	21.8 (6.0)	1.1 [−1.9, 4.2]
	@HS	35.8 (9.3)	35.5 (9.4)	0.2 [−1.1, 1.6]	-	-		-	-	
Xsens Hip	Min	−6.4 (4.1)	−3.7 (3.8)	−2.7 [−4.3, −1.2]	−2.8 (3.6)	−2.5 (3.4)	−0.4 [−1.8, 1.0]	−9.6 (4.4)	−8.6 (4.2)	−1.0 [−2.7, 0.6]
	Max	5.9 (4.5)	7.0 (4.3)	−1.1 [−2.7, 0.4]	5.0 (4.5)	5.2 (3.3)	−0.2 [−1.8, 1.5]	1.5 (5.3)	2.9 (3.3)	−1.4 [−3.3, 0.6]
	ROM	12.3 (2.7)	10.7 (2.8)	1.6 [0.5, 2.7]	7.9 (3.2)	7.6 (2.9)	0.2 [−0.7, 1.2]	11.1 (3.6)	11.4 (3.8)	−0.3 [−1.4, 0.7]
	@HS	−2.5 (4.6)	1.7 (4.6)	−4.2 [−5.8, −2.6]	-	-		-	-	
Xsens Knee	Min	−10.9 (4.9)	−13.4 (4.8)	2.5 [0.9, 4.2]	−2.1 (3.9)	−2.9 (3.5)	0.8 [−0.9, 2.5]	−7.3 (4.6)	−8.6 (4.9)	1.2 [−0.6, 3.0]
	Max	3.2 (4.0)	1.4 (3.8)	1.8 [−0.2, 3.7]	5.1 (5.0)	5.3 (4.9)	−0.1 [−2.3, 2.0]	5.4 (4.4)	5.3 (4.8)	0.1 [−1.8, 1.9]
	ROM	14.1 (3.8)	14.8 (4.2)	−0.8 [−2.2, 0.7]	7.2 (2.6)	8.1 (3.2)	−0.9 [−2.1, 0.2]	12.7 (3.3)	13.8 (3.9)	−1.1 [−2.3, 0.0]
	@HS	−4.5 (5.8)	−8.9 (5.7)	4.4 [2.2, 6.6]	-	-		-	-	
Xsens Ankle	Min	−6.2 (5.0)	−8.6 (6.0)	2.5 [0.1, 4.8]	−4.7 (5.6)	−6.2 (6.1)	1.5 [−1.0, 3.9]	−4.9 (4.1)	−7.0 (6.0)	2.1 [0.0, 4.2]
	Max	13.4 (5.4)	11.5 (7.4)	1.9 [−0.6, 4.5]	0.5 (5.6)	−0.8 (6.2)	1.3 [−1.3, 4.0]	13.5 (4.2)	12.0 (6.9)	1.5 [−1.2, 4.3]
	ROM	19.5 (4.0)	20.1 (4.9)	−0.5 [−2.3, 1.2]	5.2 (1.6)	5.3 (1.8)	−0.1 [−0.7, 0.5]	18.4 (3.6)	19.0 (3.3)	−0.6 [−2.4, 1.2]
	@HS	−2.9 (4.3)	−4.3 (5.0)	1.4 [−0.6, 3.4]	-	-		-	-	

Data for each session are mean (standard deviation). Data for the difference are difference [95% confidence interval].
